# MBX 2109, A Once-Weekly Parathyroid Hormone Replacement Therapy Prodrug: Phase 1, First-In-Human, Randomized Trial

**DOI:** 10.1210/clinem/dgae808

**Published:** 2024-11-22

**Authors:** Patricia Carney, Gordon B Cutler, Kristi Schneider, Fa Zhang, Richard DiMarchi

**Affiliations:** Clinical Operations, MBX Biosciences Inc, Carmel, IN 46032, USA; Gordon Cutler Consultancy, LLC, Deltaville, VA 23043, USA; Clinical Development, MBX Biosciences Inc, Carmel, IN 46032, USA; Department of Chemistry, Indiana University, Bloomington, IN 47405, USA; Department of Chemistry, Indiana University, Bloomington, IN 47405, USA

**Keywords:** hypoparathyroidism, parathyroid hormone, peptide therapeutics, hormone replacement therapy, prodrug, hypocalcemia

## Abstract

**Context:**

Hypoparathyroidism denotes parathyroid hormone (PTH) deficiency and impaired mineral metabolism. MBX 2109, a novel prodrug yielding a biologically active PTH peptide agonist (PTH[1-32], extended by a fatty acylated Lys^33^), is being developed as a long-acting, once-weekly PTH replacement therapy.

**Objective:**

Here, we report the safety, pharmacokinetics (PK), and pharmacodynamics (PD) of MBX 2109 in healthy volunteers.

**Methods:**

This phase 1, randomized, double-blind, placebo-controlled, multiple ascending-dose study (NCT05158335) enrolled healthy adults, who were randomly assigned 4:1 to receive MBX 2109 (200, 400, 600, and 900 μg; n = 8) or placebo (n = 2) by subcutaneous administration once weekly for 4 doses (days 1, 8, 15, and 22). The primary end point was safety and tolerability. Key secondary end points were PK and PD.

**Results:**

Overall, 40 participants (MBX 2109 n = 32, placebo n = 8) were randomly assigned (mean age, 43.3 years; 22.5% female). Treatment-emergent adverse events (TEAEs) occurred in 50% to 88% of MBX 2109 groups and in 25% of placebo participants. In the MBX 2109 groups, no severe or serious TEAEs were observed. Injection-site reaction was the most common treatment-related TEAE. The half-lives were 79 to 95 hours for MBX 2109 and 184 to 213 hours for the fatty-acylated biologically active PTH peptide, which showed dose- and time-dependent exposure increases.

**Conclusion:**

The sustained-action PTH prodrug MBX 2109 was well tolerated with no unexpected, off-target safety issues. The long half-life and flat exposure profile of MBX 2109's biologically active PTH agonist supports once-weekly administration. MBX 2109 doses were identified for future studies.

Hypoparathyroidism (hypoPT) is a rare endocrine disorder characterized by parathyroid hormone (PTH) deficiency that results in hypocalcemia, hyperphosphatemia, and low levels of 1,25-dihydroxyvitamin D (1). The altered mineral metabolism in hypoPT produces clinical manifestations in bone, kidneys, muscle, nerve, and heart that constitute a substantial burden of illness (2). Patients with hypoPT experience hypocalcemia and its attendant neuromuscular symptoms including pain, muscle cramps, numbness, and tingling (3, 4).

Compared with the general population, patients with hypoPT also experience worsened cognitive and emotional functioning including general fatigue, lack of focus (ie, brain fog), anxiety and/or depression, and other neuropsychiatric disorders (3, 5, 6).

The standard of care for hypoPT is oral supplementation with calcium, active vitamin D analogues, and magnesium (1, 2, 7). Adherence to supplements can be difficult owing to the large number of pills needed daily due to the short-lived increases in serum calcium and/or magnesium (1, 8). Large oral doses can increase fractional calcium excretion due to PTH deficiency leading to hypercalciuria, which is worsened by the 1,25-dihydroxyvitamin D–mediated increase in gastrointestinal calcium absorption (7), which increases the risk of kidney disorders (2).

Because PTH deficiency is the etiology for chronic hypoPT, hormone replacement therapy with PTH would be the ideal treatment (1, 2). However, the short half-lives (t_1/2_) of unmodified PTH(1-34) and PTH(1-84) peptides necessitate frequent administration to normalize serum calcium levels (2). For example, twice-daily subcutaneous (SC) injection of teriparatide, a recombinant PTH(1-34) analogue with a short half-life (∼1 hour) (2), was more efficacious than once-daily administration in normalizing serum calcium and reducing hypercalciuria in patients with hypoPT (9). Continuous SC (pump) infusion of PTH(1-34) normalizes both serum calcium and phosphate levels and reverses hypercalciuria in patients with hypoPT (10). Once-daily palopegteriparatide, a prodrug of PTH(1-34) conjugated to a methoxypolyethylene glycol (PEG) carrier, has demonstrated significant efficacy over placebo in patients with hypoPT with 79% vs 5%, respectively, of patients achieving a composite primary end point of normalizing serum calcium and eliminating active vitamin D and calcium supplements (8). The only recombinant DNA–derived human PTH(1-84) replacement therapy approved in the United States to treat hypoPT will be withdrawn from the market in 2024 due to manufacturing issues (11, 12). Clearly, the availability of a long-acting PTH agonist should address an unmet medical need in patients with chronic hypoPT (13).

MBX 2109 is a novel PTH peptide prodrug being developed as a long-acting, once-weekly PTH replacement therapy for hypoPT. The MBX 2109 prodrug core comprises the first 32 amino acids of human PTH extended by 2 amino acids (Sar^0^ and D-Lys^−1^) at the N terminus and 1 (Lys^33^) at the C terminus, totaling 35 amino acids. Each end of the prodrug is also extended by a covalently attached fatty C18 diacid, and the fatty acylated N-terminal dipeptide renders the prodrug biologically inactive. After SC injection, the prodrug fatty acids reversibly bind albumin. A physiologic pH- and temperature-controlled intramolecular cyclization releases the MBX 2109 fatty acylated, N-terminal dipeptide, thus converting it to the biologically active peptide, which retains the C-terminal fatty diacid at Lys^33^ (Fig. 1).

**Figure 1.**
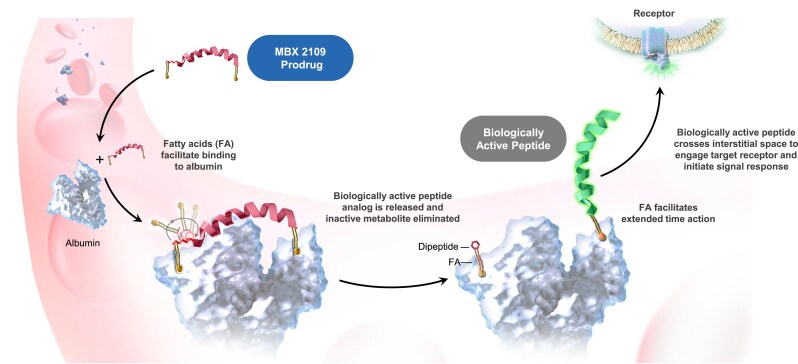
Mechanism of action of MBX 2109. MBX 2109 (prodrug) converts to biologically active peptide at a precise rate.

The objective of this phase 1, first-in-human study was to characterize the safety, pharmacokinetic (PK), and pharmacodynamic (PD) profiles of MBX 2109 administered in single and multiple ascending doses in healthy adult volunteers (ClinicalTrials.gov identifier: NCT05158335). The safety, PK, and PD profiles of multiple ascending doses of MBX 2109 are presented herein. Results from the single ascending doses of MBX 2109 will be reported separately.

## Materials and Methods

### Study Design and Ethics

This was a randomized, double-blind, placebo-controlled, single and multiple ascending-dose study. This study was conducted in accordance with the Good Clinical Practice Guideline as defined by the International Conference on Harmonisation, the Declaration of Helsinki, and/or all applicable federal and local regulations. This study was approved by all institutional review boards of the investigators’ institutions, as appropriate. All volunteers provided written informed consent before any study procedures were initiated.

### Participants

Healthy male and female adults (aged 21-60 years) with body mass index ranging from 20 through 32 kg/m^2^ were eligible for the study. All participants were required to be in good health, with women being of nonchildbearing potential (sterile or postmenopausal) and men and their partners of childbearing potential expected to use contraception.

Key exclusion criteria included any clinically significant history of active systemic disease (including but not limited to endocrine/metabolic disorders that may affect calcium levels or bone), malignancies, recent acute illness, positive results on a hepatitis panel or HIV test, abnormal vital sign measurements, or psychiatric disorders. Adults with abnormal laboratory values that suggested a clinically significant underlying disease were also excluded from participation. Exclusion criteria also included serum calcium or endogenous intact PTH(1-84) outside normal reference ranges, abnormal liver or kidney function, or a 25-hydroxyvitamin D concentration less than 20 ng/mL. Potential participants were excluded if they used medications, supplements, and other substances before and during the study that may have affected the collection or interpretation of study results or placed a participant at unnecessary risk. Calcium supplements and calcium or vitamin D–containing multivitamins were to be discontinued once eligibility was confirmed.

### Study Treatments

For the remainder of this report, the biologically inactive PTH prodrug will be described as “MBX 2109”; after release of N-terminal fatty-acylated dipeptide from the prodrug, the remaining biologically active PTH(1-32) peptide with a C-terminal fatty-acylated Lys^33^ will be called the “biologically active peptide.” The multiple-dose, sequential dose-escalation group design specified enrollment of up to 10 participants in each of 4 dose groups. In each dose group, participants were randomly assigned 4:1 to receive MBX 2109 (n = 8) or placebo (n = 2) as an SC injection in the abdomen once weekly for 4 doses (days 1, 8, 15, and 22) after an overnight fast of 8 hours or longer. MBX 2109 was stored as a frozen liquid in vials at −80 °C, at a concentration of 1 mg/mL, and thawed before injection. Matching placebo was a sterile phosphate-buffered solution. Participants were randomly assigned using a computer-generated pseudorandom permutation procedure (Fortrea).

MBX 2109 dose levels of 200, 300, 460, and 700 to 1200 μg were planned for this multiple ascending-dose study. Before each dose escalation, a safety review meeting evaluated safety and effect on serum calcium levels after 5 or more participants had been observed for 19 or more days after initial administration of that dose. Following these reviews, adjustment of higher dosages (based on accruing safety, PK, and PD data at each lower dose) resulted in administered dose levels of 200, 400, 600, and 900 μg. Participants who received all 4 weekly doses of study treatment were discharged on day 29 and those who received fewer than 4 doses of the study drug were domiciled for 96 hours or longer after their last injection of the study drug, provided there were no safety concerns requiring further observation. Participants returned for outpatient visits at 14 ± 1, 21 ± 2, and 35 ± 3 days (end-of-study visit) after the last dose of the study drug for a total study duration of approximately 13 weeks for each participant.

### Assessments

#### Safety assessments

The primary objectives of this study were to assess safety and tolerability. Safety end points included the incidence, severity, seriousness, and potential drug or drug-discontinuation relationship of treatment-emergent adverse events (TEAEs), including injection-site reactions. All TEAEs were coded using the Medical Dictionary for Regulatory Activities, version 24.1. TEAEs were defined as an event that started during or after dosing, or before dosing and increased in severity after dosing. All injection sites were assessed for pain, redness, swelling, and tenderness, each on a 0 to 4 scale with higher values reflecting a higher degree of severity.

Additionally, changes in vital sign measurements, physical examination findings, and clinical laboratory values were evaluated. Antidrug antibodies to MBX 2109 and its biologically active peptide were also analyzed using streptavidin plates (Meso Scale Discovery) coated with either the MBX 2109, its biologically active peptide, or the biotinylated linker alone as a control. Rabbit anti-MBX D1 (MBX 2109; https://www.antibodyregistry.org/AB_3291607) and rabbit anti-MBX peptide D1 (biologically active peptide; https://www.antibodyregistry.org/AB_3291606) polyclonal antibodies were used for positive controls. Gold Read buffer electrochemiluminescence was evaluated (QuickPlex SQ 120MM reader [Meso Scale Discovery]) and signal responses in coated wells were normalized to uncoated wells (linker only) for specific detection of antidrug antibodies.

#### Pharmacokinetic parameters

A key secondary objective was to evaluate the PK characteristics of the MBX 2109 prodrug and its biologically active peptide. PK end points included the determination of the following parameters for MBX 2109 and its biologically active peptide after each dose: maximum plasma concentration (C_max_), time to C_max_ (t_max_), area under the plasma concentration-time curve (AUC) to the end of the dosing period (AUC_0-τ_), time for plasma concentration to decrease by 50% (t_1/2_), apparent total clearance (CL/F), apparent volume of distribution during the terminal elimination phase (V_z_/F), and apparent volume of distribution estimated for steady state (V_SS_/F). In addition, C_max_ and AUC values after the first and last doses were analyzed to assess the relationship between exposure and dose administered, and to determine the degree of drug accumulation.

Blood samples for assessment of PK parameters were drawn predose and at 2, 4, 8, 12, 24, 48, 60, 72, 84, 96, 120, and 144 hours post dose on days 1, 8, 15, and 22. After day 22, or for any participants who discontinued the study before day 22, additional samples were drawn at 168, 336, 504, and 840 hours post dose.

#### Pharmacodynamic parameters

Other key secondary objectives were to evaluate the following PD parameters: serum (absolute) calcium; albumin-adjusted serum calcium (adj-Ca), maximum observed effect of adj-Ca (E_max, adj-Ca_) and baseline-adjusted area under the effect-time curve during a dosing interval (AUEC_0-τ, adj_); phosphate; 1,25-dihydroxyvitamin D; 25-hydroxyvitamin D; magnesium; intact PTH(1-84); C-telopeptide of type I collagen (CTx); procollagen 1 intact N-terminal propeptide (P1NP); and bone-specific alkaline phosphatase (BSAP). Total 24-hour urinary calcium, phosphate, magnesium, urinary fractional calcium excretion, and calcium:creatinine ratios were also evaluated.

Blood samples for assessment of serum calcium, phosphate, and albumin were drawn twice on day −1 before baseline (morning and 12 hours later), and then at baseline (pre dose) and at 24, 48, 60, 72, 84, 96, 120, and 144 hours post dose on days 1, 8, 15, and 22, and again, 14 and 21 days after the last dose. Serum endogenous PTH(1-84); 1,25-dihydroxyvitamin D; 25-hydroxyvitamin D; magnesium; CTx; P1NP; and BSAP levels were determined on days 1, 4, 12, 19, 25, and 29, and again 14 days (plus 21 days for PTH[1-84]) after the last dose. A 24-hour urine collection was completed before randomization and on days 11, 18, and 25 to monitor calcium excretion.

### Statistical Analysis

As an exploratory qualitative study, no prespecified inferential hypothesis testing was performed. Thus, a determination of sample size was not based on statistical power. Rather, the planned dose-level cohorts of 10 participants were considered sufficient to evaluate the safety, PK, and PD effects of MBX 2109. The safety population included all participants who received 1 or more doses of study medication (MBX 2109 or placebo). Safety data were summarized descriptively including counts and percentages. PK parameters were determined from plasma concentrations of MBX 2109 and biologically active peptide using standard noncompartmental methods. The dose-exposure relationships for MBX 2109 and its biologically active peptide were examined for individual participants and across dose groups. The PK analysis was conducted by Fortrea using Phoenix WinNonlin version 8.3.5 (Certara). PD parameters were summarized descriptively and absolute change and percentage change from baseline values were calculated. The PK and PD populations included all participants who received 1 or more doses of MBX 2109 and had 1 or more valid PK or PD assessments.

## Results

### Participants

This first-in-human study was completed between November 10, 2021, and May 31, 2023, at 2 clinical research units in the United States. Of 40 participants randomly assigned, a total of 32 received MBX 2109 and 8 received placebo in the 200-, 400-, 600-, and 900-µg dose cohorts (MBX 2109 n = 8, placebo n = 2 at each dose level). One participant in the MBX 2109 400-μg group was lost to follow-up and 1 participant in the 900-μg group withdrew from the study. Therefore, the safety population included 40 participants, and the PK and PD populations each included 38 participants. Demographic and baseline characteristics were similar across groups (Table 1). The mean (SD) age was 43.3 (10.2) years, 22.5% (9/40) of participants were female, and the mean (SD) body mass index was 26.3 (2.2).

**Table 1.** Baseline demographics and characteristics

Characteristic	MBX 2109				
	200 µg (n = 8)	400 µg (n = 8)	600 µg (n = 8)	900 µg (n = 8)	Placebo (n = 8)
Age, mean (SD), y	41.5 (11.3)	41.4 (9.0)	43.3 (11.7)	47.4 (5.5)	42.9 (13.3)
Sex, n (%)					
Female	2 (25.0)	2 (25.0)	1 (12.5)	3 (37.5)	1 (12.5)
Male	6 (75.0)	6 (75.0)	7 (87.5)	5 (62.5)	7 (87.5)
Race, n (%)					
White	6 (75.0)	2 (25.0)	3 (37.5)	4 (50.0)	7 (87.5)
Black	2 (25.0)	5 (62.5)	4 (50.0)	4 (50.0)	1 (12.5)
Other or multiple	0	1 (12.5)	1 (12.5)	0	0
Ethnicity, n (%)					
Hispanic or Latino	3 (37.5)	3 (37.5)	1 (12.5)	1 (12.5)	3 (37.5)
BMI, mean (SD)	26.7 (1.7)	27.7 (1.9)	27.1 (1.8)	24.9 (2.6)	25.2 (1.9)

Abbreviation: BMI, body mass index.

#### Safety assessments

Overall, 55% of participants reported TEAEs (placebo 25%, MBX 2109 ranging from 50% to 87.5% across dose groups). Most TEAEs were mild and 2 were moderate. One severe TEAE (plasma cell myeloma) occurred in a participant receiving placebo (Table 2). There were no serious TEAEs or deaths. Six participants had dosing discontinued due to TEAEs with 4 due to TEAEs of COVID-19/positive SARS-CoV-2 test and 2 due to TEAEs of hypercalcemia (1 each in the 600- and 900-µg MBX 2109 dose groups). Injection-site reaction was the most common treatment-related TEAE with no patients in the placebo group and 10 (31.3%) in MBX 2109 dose groups. Injection-site reactions were not dose related and were generally mild in intensity, red with a diameter of less than 50 mm, flat, and resolved within 3 days without intervention. Treatment-related TEAEs of hypercalcemia occurred in the 600-µg (n = 1) and 900-µg (n = 2) MBX 2109 dose groups. None of the TEAEs of hypercalcemia were symptomatic; the maximal serum calcium level was 11.4 mg/dL or less and all resolved without intervention. There was no imbalance among treatment groups in potentially vasodilatory-related TEAEs.

**Table 2.** Adverse event summary

Parameter	MBX 2109				
	200 µg (n = 8)	400 µg (n = 8)	600 µg (n = 8)	900 µg (n = 8)	Placebo (n = 8)
Any TEAE	4 (50.0)	4 (50.0)	7 (87.5)	5 (62.5)	2 (25.0)
Mild	4 (50.0)	4 (50.0)	7 (87.5)	4 (50.0)	2 (25.0)
Moderate	0	0	0	2 (25.0)	0
Severe	0	0	0	0	1 (12.5)
Treatment-related TEAE	3 (37.5)	3 (37.5)	5 (62.5)	3 (37.5)	0
Mild	3 (37.5)	3 (37.5)	5 (62.5)	3 (37.5)	0
Moderate	0	0	0	1 (12.5)	0
Severe	0	0	0	0	0
TEAE leading to study drug discontinuation	1 (12.5)	2 (25.0)	1 (12.5)	1 (12.5)	1 (12.5)
Most common TEAEs*^a^*					
Injection-site erythema	0	2 (25.0)	2 (25.0)	2 (25.0)	0
SARS-CoV-2 test positive	0	3 (37.5)	1 (12.5)	0	1 (12.5)
Hypercalcemia	0	0	1 (12.5)	2 (25.0)	0
Constipation	0	0	0	2 (25.0)	1 (12.5)

Abbreviation: TEAE, treatment-emergent adverse event.

^*a*^Occurring in 2 or more participants in any treatment group.

There were no clinically significant changes in vital sign measurements, findings on physical examination, or findings on routine clinical laboratory assessments, other than the expected increases in serum calcium and urine calcium levels in participants treated with MBX 2109. A low incidence of antidrug antibodies (generally of low titer) to MBX 2109 or its biologically active peptide was seen, and there were no apparent clinical sequelae in participants positive for antidrug antibodies.

#### Pharmacokinetic assessments

**Figure 2.**
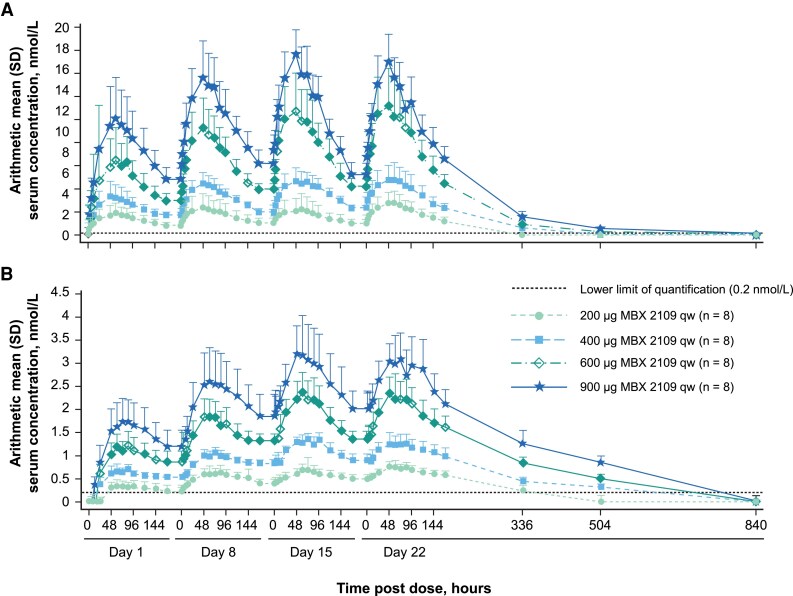
Pharmacokinetic profile of MBX 2109 after multiple ascending doses. (A) Prodrug. (B) Biologically active drug. MBX 2109 prodrug and biologically active drug plasma concentrations increased linearly and dose proportionally with each weekly dose. Data are arithmetic mean ± SD. Abbreviation: qw, once weekly.

Plasma PK parameters for MBX 2109 and its biologically active peptide are summarized in Table 3 and in Fig. 2. The median t_max_ values ranged across MBX 2109 doses from 48.0 to 60.0 hours. The geometric mean elimination t_1/2_ values ranged from 78.8 to 94.9 hours. Increases in MBX 2109 systemic exposure were dose proportional. As assessed from the geometric coefficient of variation (CV), between-participant variability in MBX 2109 AUCs and C_max_ was high at the 200-µg dose level (45.3%-68.8%) and was generally moderate at the 400- to 900-µg dose levels (12.7%-43.1%). On day 22, no consistent trends were observed in geometric mean CL/F, V_z_/F, and V_SS_/F values for MBX 2109 across doses (data not shown).

**Table 3.** Pharmacokinetic parameters

	MBX 2109 once-weekly dose			
	200 µg (n = 8)	400 µg (n = 8)	600 µg (n = 8)	900 µg (n = 8)
MBX 2109 parameter, geometric mean (CV)				
Day 1				
AUC_0-τ_, days•nmol/L	222 (62.5)	432 (36.6)	817 (36.3)	1320 (30.1)
C_max_, nmol/L	1.89 (67.9)	3.59 (43.1)	7.17 (40.6)	11.1 (31.1)
t_max_, h*^a^*	60.0 (48.0, 96.0)	54.0 (48.0, 120)	72.0 (60.0, 84.1)	60.0 (60.0, 72.3)
Day 22				
AUC_0-τ_, days•nmol/L	365 (45.3)	696 (26.0)	1500 (28.2)	2020 (14.2)
C_max_, nmol/L	2.91 (52.8)	5.35 (29.2)	12.6 (30.5)	17.1 (12.7)
t_max_, h*^a^*	48 (47.9, 60)	60.0 (8.0, 72.1)	48.3 (24.1, 60.0)	48.0 (48.0, 60.0)
t_1/2_, h	91.7 (41.3)	94.9 (27.0)	78.8 (6.5)	85.2 (2.9)
C_min_, nmol/L	1.07 (45.6)	2.48 (9.6)	4.53 (15.9)	5.77 (20.7)
AR_Cmax_	1.62 (22.6)	1.53 (25.5)	1.76 (22.1)	1.63 (16.3)
AR_AUC_	1.72 (21.3)	1.63 (25.8)	1.84 (15.3)	1.60 (14.3)
Peak-to-trough ratio	2.67 (18.5)	2.02 (28.0)	2.70 (39.2)	2.95 (15.2)
Biologically active peptide parameter, geometric mean (SD)				
Day 1				
AUC_0-τ_, days•nmol/L	47.4 (17.4)	82.9 (19.0)	145 (22.0)	212 (29.2)
C_max_, nmol/L	0.398 (24.7)	0.697 (16.8)	1.26 (25.1)	1.77 (29.2)
t_max_, h*^a^*	90.0 (48.0, 168)	84.0 (48.0, 168)	84.0 (60.0, 120)	84.1 (60.0, 120)
Day 22				
AUC_0-τ_, days•nmol/L	108 (14.5)	186 (15.6)	322 (17.2)	445 (18.3)
C_max_, nmol/L	0.783 (17.5)	1.30 (17.6)	2.39 (19.5)	3.13 (17.0)
t_max_, h*^a^*	60.0 (48.0, 84.0)	60.1 (24.0, 84.4)	60.0 (48.0, 84.2)	59.8 (48.0, 72.2)
t_1/2_, h*^a^*	213 (39.1)	190 (22.0)	184 (10.4)	204 (6.6)
C_min_, nmol/L	0.465 (17.6)	0.799 (15.7)	1.27 (13.5)	1.89 (19.6)
AR_Cmax_	1.94 (21.6)	1.87 (19.3)	1.92 (9.3)	1.99 (11.4)
AR_AUC_	2.43 (17.1)	2.25 (19.1)	2.22 (8.9)	2.36 (7.4)
Peak-to-trough ratio	1.60 (15.8)	1.47 (12.6)	1.79 (20.8)	1.59 (5.1)

Abbreviations: AR_AUC_, accumulation ratio for AUC; AR_Cmax_, accumulation ratio for C_max_; AUC_0-τ_, area under concentration-time curve to end of the dosing period; C_max_, maximum plasma concentration; C_min_, minimum plasma concentration; t_1/2_, time for plasma concentration to decrease by 50%; t_max_, time to reach maximum plasma concentration.

^*a*^T_max_ values are presented as the median (minimum, maximum).

The biologically active peptide appeared in plasma with median t_max_ values ranging from 54.0 to 72.1 hours. Geometric mean peak-to-predose trough concentration ratios after the fourth dose ranged from 1.47 to 1.79 for the 4 dose strengths, indicating that the weekly dosing regimen achieved consistent levels of the biologically active peptide between weekly injections. After reaching C_max_, plasma concentrations of the biologically active peptide slowly declined, with geometric mean t_1/2_ values ranging from 184 to 213 hours. In general, increases in systemic exposure of the biologically active peptide were dose proportional. After multiple weekly doses, accumulation of the biologically active peptide in plasma was observed on days 8, 15, and 22 (compared with day 1) across all dose levels with the incremental increase decreasing with each dose (see Fig. 2). Between-participant variability in the biologically active peptide AUC_0-τ_ and C_max_ was low to moderate, with geometric CV values ranging from 9.0% to 29.2% and 9.5% to 29.5%, respectively. On day 22 after the fourth dose administration, geometric mean values for the ratios of biologically active peptide to prodrug based on AUC_0-τ_ ranged from 0.215 to 0.296 with no discernable trend across the MBX 2109 dose level.

#### Pharmacodynamic assessments

**Figure 3.**
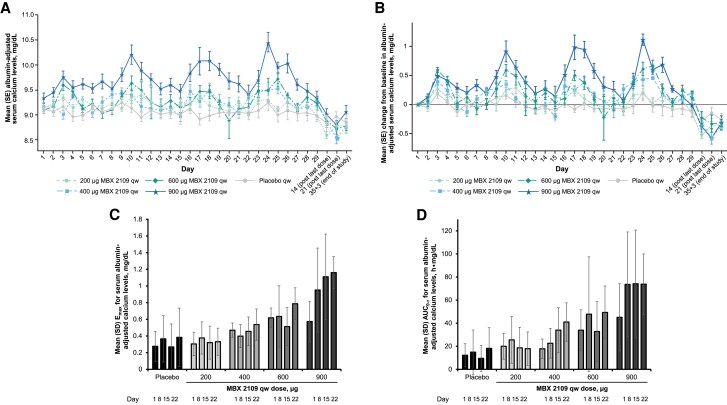
Albumin-adjusted serum calcium levels after multiple ascending doses of MBX 2109. (A) Mean ± SE concentration over time. (B) Mean ± SE change from baseline using maximum change across morning and evening time points. (C) Mean ± SD maximum observed effect. (D) Mean ± SD AUC_0-τ_. Dose-related increases in mean albumin-adjusted serum calcium were observed. Abbreviations: AUC_0-τ_, area under the effect-time curve during a dosing interval; Emax, maximum observed effect; qw, once weekly.

For the 600- and 900-μg MBX 2109 dose groups, peak adj-Ca levels were generally observed 48 hours after injection, while peak levels were generally achieved 48 to 72 hours after injection in the 400- and 200-μg dose groups (Fig. 3A and 3B). The mean adj-Ca for all dose groups remained within a normal range for serum calcium (8.5-10.5 mg/dL) (see Fig. 3A). In the 600- and 900-µg MBX 2109 dose groups, increases in adj-Ca were evident in the week after the first injection (Fig. 3C). For all dose groups, adj-Ca levels declined from their peak toward their baseline levels before the next weekly injection (see Fig. 3A and 3B). After the last injection, serum calcium levels decreased to slightly below baseline in all treatment groups. Dose-dependent and time-related increases in E_max, adj-Ca_, relative to placebo, at morning and evening time points, were seen at weekly MBX 2109 doses of 400 µg or greater (see Fig. 3C). E_max, adj-Ca_ results were similar in placebo and MBX 2109 200-µg dose groups (see Fig. 3C). Changes from baseline in arithmetic mean E_max, adj-Ca_ after the fourth weekly dose were 0.537, 0.787, and 1.16 mg/dL at the 400-, 600-, and 900-µg MBX 2109 dose levels, respectively, and were greater than for the placebo (0.383 mg/dL) or the 200-µg MBX 2109 dose groups (0.331 mg/dL). In the 900-µg MBX 2109 dose group, increases in E_max, adj-Ca_ were near maximal after the second injection. A similar dose-response relationship to that seen using E_max, adj-Ca_ was observed using the average adj-Ca over the dosing interval (AUEC_0-τ, adj_ [h • mg/dL]: MBX 2109 200 µg, 17.9; 400 µg, 41.0; 600 µg, 49.3; 900 µg, 73.8; and placebo 18.2; Fig. 3D).

**Figure 4.**
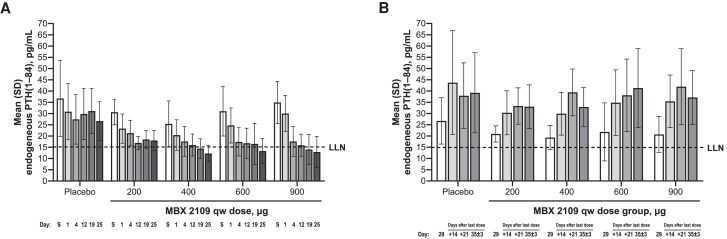
Endogenous serum PTH(1-84) level after multiple ascending doses of MBX 2109. (A) MBX 2109 was associated with dose-related decreases in mean intact PTH(1-84) levels, which were suppressed below the lower limit of the reference range. (B) After the last injection, endogenous PTH(1-84) levels rose in all treatment groups to slightly above baseline levels. Abbreviations: LLN, lower limit of normal; PTH, parathyroid hormone; qw, once weekly; S, screening.

At all doses studied, MBX 2109 administration decreased endogenous PTH(1-84) levels, relative to placebo, in a dose-dependent fashion (Fig. 4A). For participants who received MBX 2109 in 400-, 600-, and 900-µg doses, endogenous PTH(1-84) levels were generally suppressed below the lower limit of the reference range (15 pg/mL). Decreases in endogenous PTH(1-84) were apparent at all MBX 2109 doses a day after the initial administration and were near maximal before the second injection. After the last injection in all treatment groups, endogenous PTH(1-84) levels rose in all treatment groups to slightly above baseline levels (Fig. 4B). Decreases in 1,25-dihydroxyvitamin D levels, relative to placebo, occurred in all MBX 2109 dose groups (Supplementary Fig. S1) (14). The decreases were near maximum on day 4 after the initial administration at MBX 2109 doses of 400 µg or greater. After the fourth dose, 1,25-dihydroxyvitamin D levels increased to or were slightly above baseline levels in all MBX 2109 groups.

**Figure 5.**
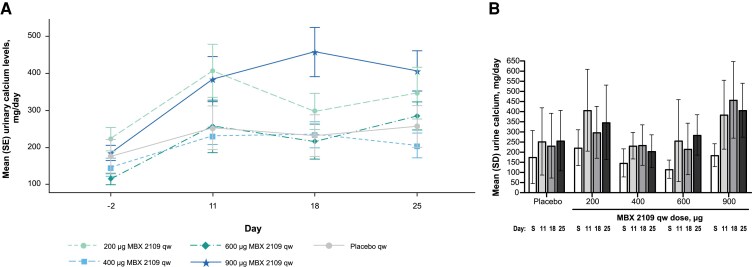
Urinary calcium excretion levels after multiple ascending doses of MBX 2109. (A) Mean ± SE urinary calcium excretion over time. (B) Mean ± SE change from baseline in urinary calcium excretion. Mean urinary calcium levels increased after the first dose of MBX 2109; levels continued to increase with 900 μg MBX 2109 and remained stable in other dose groups. Abbreviations: qw, once weekly; S, screening.

Twenty-four–hour urinary calcium excretion increased, relative to placebo, in the 900-µg MBX 2109 dose group but not in the lower MBX 2109 dose groups (Fig. 5A and 5B). Increases in urinary calcium:creatinine ratio and urinary fractional calcium excretion were also seen in the MBX 2109 900-µg dose group but not in the other MBX 2109 dose groups or in the placebo group (data not shown). Serum magnesium levels, phosphate levels, 25-hydroxyvitamin D levels, and urinary phosphate and magnesium excretion were not affected by MBX 2109 treatment (data not shown).

**Figure 6.**
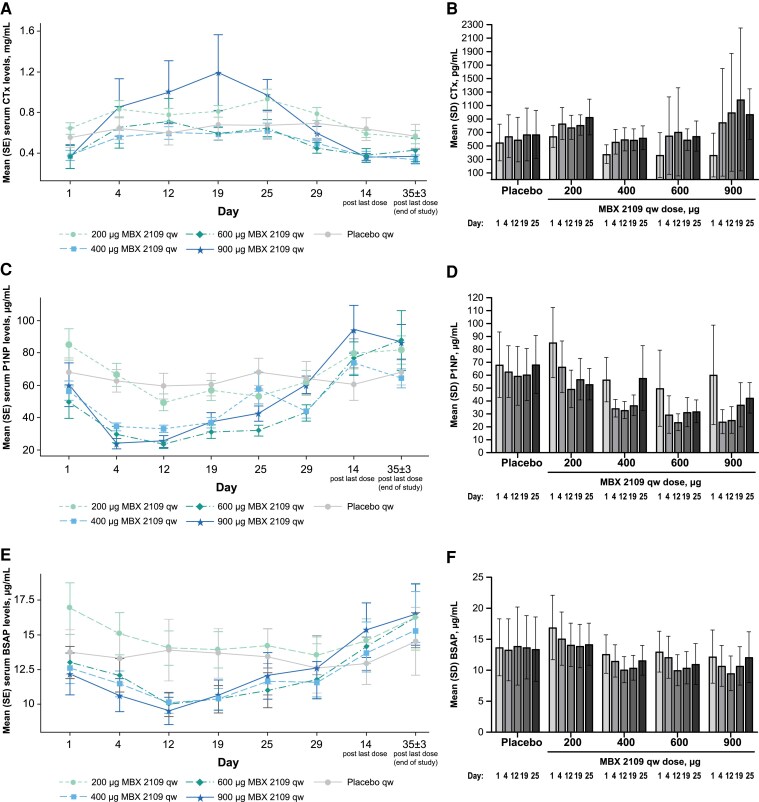
Serum CTx, P1NP, and BSAP levels. (A) Mean ± SE serum CTx levels over time. (B) Mean ± SE change from baseline in CTx levels. (C) Mean ± SE serum P1NP levels over time. (D) Mean ± SE change from baseline in P1NP levels. (E) Mean ± SE serum BSAP levels over time. (F) Mean ± SE change from baseline in BSAP levels. CTx levels slightly increased in all MBX 2109 groups after the first dose; minimal changes were observed with subsequent MBX 2109 doses except the 900-μg dose. P1NP and BSAP levels transiently decreased with repeated MBX 2109 dosing and returned toward baseline levels after dosing. Abbreviations: BSAP, bone-specific alkaline phosphatase; CTx, C-telopeptide of type 1 collagen; EOS, end of study; P1NP, procollagen 1 intact N-terminal propeptide; qw, once weekly.

In response to MBX 2109 treatment, CTx levels increased in a dose-dependent fashion with increases apparent on day 4 after the initial administration (Fig. 6A and 6B). P1NP and BSAP levels decreased in a non–dose-dependent fashion with maximal decreases seen between days 4 and 12 followed by levels increasing by day 19 and nearly to baseline values by day 25 (Figs. 6C-6F, respectively).

## Discussion

The key objectives in this first-in-human, placebo-controlled, randomized, double-blind study conducted in healthy participants were to evaluate the safety and tolerability of MBX 2109, to determine if the PK profile of its biologically active peptide would support a once-weekly dosing regimen, and to identify pharmacologic MBX 2109 doses to evaluate in future studies conducted in patients with hypoPT.

In the multiple ascending-dose portion of the study, there were no severe or serious TEAEs; the only TEAE occurring in 3 or more participants was COVID-19 infection. Hypercalcemia was observed in the MBX 2109 600-μg (n = 1) and 900-μg (n = 2) cohorts. These hypercalcemia TEAEs were asymptomatic with the maximal serum calcium level of 11.4 mg/dL or less and resolved without intervention. Hypercalcemia was an expected, on-target outcome in a study attempting to define a maximally tolerated dose of a PTH agonist. Vasodilation-mediated TEAEs, such as orthostatic hypotension, are a potential complication of treatment with a PTH agonist (15, 16). No imbalance between placebo and MBX 2109 groups in TEAEs possibly associated with vasodilation was noted in this study. Injection-site reaction TEAEs were more common in the MBX 2109 groups than in the placebo group. The reactions were generally mild in intensity, presenting as red and flat, less than 50 mm in diameter, and resolving spontaneously. No clinically significant changes in vital sign measurements, physical examinations, or routine clinical laboratory values (other than the expected increases in serum and urine calcium) were seen with MBX 2109 treatment.

MBX 2109 is designed as a prodrug that undergoes intramolecular cyclization to release the biologically active peptide containing the PTH(1-32) sequence, which is reversibly bound to albumin through a C-terminal fatty acylated Lys^33^. This prodrug design and reversible binding to albumin yielded a biologically active peptide with a half-life between 184 and 213 hours, which supports a once-weekly dosing regimen. Exposure to the biologically active peptide increased in a dose- and time-dependent fashion. While complete steady-state exposure to the biologically active peptide is predicted to occur after 5 to 6 weekly MBX 2109 injections, the incremental increases in exposure to the biologically active peptide decreased after each injection with most of the incremental increase in exposure seen after the second or third weekly injection. Due to the long half-life, the peak-to-trough exposures to the biologically active peptide with weekly dosing were relatively flat with ratios ranging from 1.47 and 1.79 across dose levels. The flat exposure profile could translate into lower fluctuations in serum calcium and fewer symptoms due to hypercalcemia and hypocalcemia, compared with PTH agonists with a shorter half-life. Additionally, variability in the biologically active peptide in AUC_0-τ_ and C_max_ was low to moderate.

A dose- and time-dependent increase in the maximal serum calcium response and decrease in endogenous PTH(1-84) levels were seen with MBX 2109 treatment. In healthy participants, PTH agonist-induced increases in serum calcium are expected to lead to negative feedback on the intact parathyroid glands to reduce secretion of endogenous PTH(1-84). The decreases in endogenous PTH(1-84) occurred with lower MBX 2109 doses (200 µg), relative to doses that increased serum calcium levels (≥400 µg).

The reason for these differences in dose dependency for the serum calcium increase may be due to counterregulatory responses to maintain serum calcium homeostasis during PTH agonist–mediated serum calcium increases in these healthy participants. This may obscure the ability to observe the full effect of MBX 2109 to raise serum calcium. For example, elevated serum calcium levels have been shown to activate calcium-sensing receptors, thereby inhibiting endogenous PTH release from the parathyroid gland and stimulating calcitonin (a calcium-lowering hormone) release from the thyroid (17). In addition, fibroblast growth factor-23 released from bone can bind to the fibroblast growth factor receptor and the coreceptor klotho to inhibit phosphate resorption in the kidney, suppress synthesis of 1,25 dihydroxyvitamin D, and decrease PTH secretion from the parathyroid gland (2, 18). These counterregulatory mechanisms of calcium homeostasis may work against the active peptide of MBX 2109, which could explain the lowered trough concentrations of serum calcium between weekly doses of MBX 2109.

In the posttreatment follow-up (35 ± 3 days after the last MBX 2109 dose), serum calcium was lower than baseline, while endogenous PTH(1-84) increased above baseline levels. The decline of the active peptide of MBX 2109 may have led to this observed decline in serum calcium, which in turn elevated endogenous PTH to restore baseline calcium levels. Notably, however, the serum calcium levels remained within the normal physiologic range (8.5-10.5 mg/dL) throughout follow-up (19).

Future research will be needed to elucidate whether the homeostatic mechanisms that resulted in fluctuations toward lower serum calcium during the weekly interdose intervals in MBX 2109–treated healthy volunteers may instead help to restore serum calcium closer to normal levels in patients with hypoPT. Because patients with hypoPT will have lower basal levels of PTH than healthy individuals, they are less likely to reach serum calcium levels that would activate downward calcium-regulatory responses. We hypothesize that this will lead to smaller weekly interdose serum calcium fluctuations than were seen in healthy volunteers. Nonetheless, the PD parameters in healthy participants have identified a range of pharmacologically biologically active MBX 2109 doses.

Acute treatment of healthy participants or postmenopausal women with PTH is associated with increases, evident within the first month of treatment, in markers of bone formation (P1NP and BSAP) and resorption (CTx) (20, 21). MBX 2109 increased CTx in a time- and dose-dependent fashion. In contrast, P1NP and BSAP decreased in a non–dose-dependent fashion within 4 days after the initial MBX 2109 injection. However, after the third weekly MBX 2109 injection, bone formation markers began to increase and were at or near baseline levels at day 25 after the fourth MBX 2109 injection. The mechanism of the transient decrease followed by a return to near or at baseline markers of bone formation is unknown. The clinical relevance of these changes in markers of bone turnover to patients with hypoPT is questionable. Unlike healthy participants, patients with hypoPT have high bone mineral density with low levels of bone turnover (22). Longer-term studies of patients with hypoPT treated with PTH agonists have demonstrated increases in markers of bone formation and resorption accompanied by reductions, within the normal range, in bone mineral density (23).

In these healthy participants, MBX 2109 treatment was associated with non–dose-dependent, acute decreases in serum 1,25-dihydroxyvitamin D, which were seen by day 4 after the initial injection and were generally maintained while participants were receiving treatment. Levels of 25-hydroxyvitamin D were not affected by MBX 2109 treatment. As an established action of PTH is to increase 1,25-dihydroxyvitamin D levels by stimulating expression of 25-hydroxyvitamin D 1α-hydroxylase (1), the decrease in 1,25-dihydroxyvitamin D levels with MBX 2109 was unexpected. By acting through the calcium-sensing receptor, hypercalcemia can lead to negative feedback on 25-hydroxyvitamin D 1α-hydroxylase activity (24). However, the different dose- and time-dependent relationships between the effects of MBX 2109 on serum calcium and 1,25-dihydroxyvitamin D levels do not support increases in serum calcium as being the sole explanation for this finding. The high baseline 1,25-dihydroxyvitamin D levels in the MBX 2109 groups decreased at the first postrandomization visit and remained relatively constant thereafter, suggesting that the observed decreases may be explained, in part, by a regression to the mean. The cause for this finding and its clinical relevance to patients with hypoPT are unknown. Patients with chronic hypoPT treated for 6 months with a PTH agonist maintained normal 1,25-dihydroxyvitamin D levels, despite cessation of active vitamin D supplements (25).

This study has several limitations. Only a small number of participants were studied, which did not allow for inferential statistical analysis. The enrolled participants in this study were predominantly male, which may limit the generalizability to women. Unlike patients with chronic hypoPT, the heathy participants in this study had intact parathyroid glands and normal calcium homeostasis, which confounds direct extrapolation of results to patients with chronic hypoPT. Given the differences between healthy participants and patients with chronic hypoPT in rates of bone turnover as well as the brief treatment period in this study, longer-term studies are needed to establish the effect of MBX 2109 on bone health in patients with hypoPT. Ongoing and future studies will evaluate the safety, PK, and efficacy of MBX 2109 in patients without hypoPT who have normal and impaired renal function (phase 1, NCT06496217) and in patients with hypoPT and normal renal function (phase 2, NCT06465108).

### Conclusions

In the multiple ascending-dose portion of this first-in-human, randomized, double-blind study, MBX 2109, a peptide prodrug yielding a biologically active sustained action PTH agonist, was well tolerated with no unexpected, off-target safety issues noted. With weekly injections, dose-proportional increases in MBX 2109 and its resultant biologically active peptide were observed. The long half-life of the biologically active peptide supports once-weekly administration while maintaining a flat exposure profile exemplified by a low peak-to-trough ratio over the course of a week. Pharmacologically biologically active MBX 2109 doses that increased serum calcium levels in a dose- and time-related fashion were identified for advancement into future studies in patients with chronic hypoPT.

## Data Availability

MBX Biosciences is committed to providing access to deidentified, patient-level clinical trial data; study reports; study protocols; and statistical analysis plans to qualified researchers on reasonable request. Requests for study data may be submitted via email to info@mbxbio.com. Requesters should include a description of how the data will be used in their research, their plans for dissemination of the data in the medical literature, and details about their research team. MBX Biosciences retains the right to approve or reject any request at its sole discretion. If approved, the requestor will be required to enter into a data use agreement with MBX Biosciences. Data requests will be considered for up to 12 months from the publication date of this manuscript.

## Clinical Trial Information

ClinicalTrials.gov registration number NCT05158335 (registered December 2, 2021).

## Abbreviations

adj-Ca: albumin-adjusted serum calcium
AUC: area under plasma concentration-time curve
AUC_0-τ_: AUC to the end of the dosing period
AUEC_0-τ,adj_: baseline-adjusted AUC_0-τ_
BSAP: bone-specific alkaline phosphatase
CL/F: apparent total clearance
C_max_: maximum plasma concentration
CTx: C-telopeptide of type I collagen
CV: coefficient of variation
E_max, adj-Ca_: maximum observed effect of adj-Ca
hypoPT: hypoparathyroidism
P1NP: procollagen 1 intact N-terminal propeptide
PD: pharmacodynamics
PK: pharmacokinetics
PTH: parathyroid hormone
SC: subcutaneous
t_1/2_: time for plasma concentration to decrease by 50%
TEAE: treatment-emergent adverse events
t_max_: time to C_max_
V_SS_/F: apparent volume of distribution estimated for steady state
V_z_/F: apparent volume of distribution during terminal elimination phase
