# Correction to: “MBX 2109, A Once-Weekly Parathyroid Hormone Replacement Therapy Prodrug: Phase 1, First-In-Human, Randomized Trial”

**DOI:** 10.1210/clinem/dgae889

**Published:** 2025-01-13

In the above-mentioned article by Carney P, Cutler GB, Schneider K, Zhang F, and DiMarchi R (*J Clin Endo Metab.* 2024; doi: 10.1210/clinem/dgae808), there were errors in the affiliations and the Disclosures section.

In the originally published article, the affiliations for Richard DiMarchi were listed as “Clinical Development, MBX Biosciences Inc, Carmel, IN 46032, USA” (affiliation 3) and “Co-Founder, MBX Biosciences Inc, Carmel, IN 46032, USA” (affiliation 5). Affiliation 3 was incorrectly attributed to Richard DiMarchi; therefore, the superscript “3” has been removed from the author’s name in the byline. The publisher acknowledges the error. Because affiliation 5 is not considered an affiliation by the journal, it has been deleted. In the corrected article, affiliation 4, “Department of Chemistry, Indiana University, Bloomington, IN 47405, USA,” is attributed to Richard DiMarchi. The publisher acknowledges omitting the Indiana University affiliation provided for Richard DiMarchi.

In the originally published article, in the Disclosures section, the fourth sentence read “R.D. is a cofounder and director of MBX Biosciences.” Because the author was not the director of MBX Biosciences at the time of article publication, the word “former” has been added. In the corrected article, the sentence now reads “R.D. is a cofounder and former director of MBX Biosciences.”

The article has been corrected online.

doi: 10.1210/clinem/dgae808

